# Bilateral Internal Thoracic Artery Grafting in Coronary Surgery: A Three-Tier Decision Framework

**DOI:** 10.3390/jcm15207932

**Published:** 2026-10-14

**Authors:** Gabriele Di Giammarco, Massimo Massetti

**Affiliations:** 1 Dipartimento di Neuroscienze, Imaging e Scienze Cliniche, Cattedra di Cardiochirurgia, Università “G. d’Annunzio”, 66100 Chieti, Italy; 2 Dipartimento di Malattie Cardiovascolari, Cattedra di Cardiochirurgia, Università Cattolica del Sacro Cuore, 00168 Roma, Italy; massimo.massetti@unicatt.it

**Keywords:** aortocoronary bypass, bilateral internal mammary artery, arterial revascularisation, deep sternal infection, skeletonization, competitive flow, coronary-subclavian steal

## Abstract

The left internal mammary artery grafted to the left anterior descending artery (LAD) is the main determinant of survival in coronary artery bypass grafting, but the choice of the second graft remains controversial. The use of both mammary arteries offers a greater long-term survival benefit compared with a single mammary artery graft; however, this advantage is delayed and diminished when compared with a single mammary artery graft plus a radial artery graft. Furthermore, this approach is associated with a higher risk of sternal complications and, via mechanisms of competitive flow and steal phenomenon, of perioperative coronary malperfusion. This article proposes organising the clinical decision-making process according to three hierarchical levels: (1) absolute contraindications based on the patient’s medical history that preclude the use of a particular graft (thoracic irradiation, subclavian artery disease, acute aortic dissection); (2) the haemodynamic adequacy of the graft for the target coronary territory; (3) the assessment of the risk of delayed sternal healing in the individual patient, where skeletonization has reduced the historical significance of diabetes. Overlaying these is a transversal variable represented by the harvesting technique (pedicled mammary artery vs. skeletonized mammary artery, together with the surgeon’s learning curve), which re-interprets apparently unfavourable results and distinguishes the risk associated with the graft itself from that of the surgical procedure. The model aims to rationalise patient selection and reduce the persistent underutilisation of bilateral mammary artery grafting. It should be noted at the outset that this survival advantage rests largely on observational and propensity-matched evidence and has not been confirmed on an intention-to-treat basis by the only adequately powered randomised trial, so that the model proposed here is offered as a framework for selection rather than as a claim of established superiority.

## 1. Introduction

The view that the left internal mammary artery anastomosed to the LAD is the single most important determinant of survival following coronary artery bypass grafting is one of the few assumptions in coronary surgery that is universally accepted. On the second graft, however, consensus breaks down. The bilateral internal mammary artery (BIMA) is associated, in the observational literature, with the best long-term survival rates among the available options, yet it is used in only a small minority of procedures—less than 5 per cent in the United States and less than 10 per cent in Europe [1,2]. The reason for this underutilisation is not inertia, but the nature of the benefit: real but delayed over time, set against an immediate risk of complications—sternal and haemodynamic—including perioperative mortality. This article proposes breaking down the decision into three hierarchical levels, which the surgeon goes through in sequence.

## 2. From Vein to Artery: The Trajectory of the Evidence

The history of the bilateral mammary bypass is, to a large extent, the story of a benefit that took decades to become apparent. The starting point was 1986, when the Cleveland Clinic demonstrated—by comparing 2306 patients with a mammary bypass graft to the LAD and 3625 patients revascularised with a saphenous vein graft (SVG) alone—that the arterial graft reduced ten-year mortality, reinfarction, rehospitalisation for cardiac events and the need for reoperation [3]. That finding, which transformed the LIMA from a technical option into the standard of care, immediately raised the next question: if one mammary artery graft improves survival, do two improve it further? The answer from the same institution came thirteen years later, in the form of a propensity-matched comparison involving over ten thousand patients: two mammary arteries were better than one, but the survival curves diverged in the second post-operative decade, not the first [4]. The twenty-year follow-up, involving 1152 matched pairs, confirmed and amplified this phenomenon: the difference grew progressively towards the twentieth year, and the benefit was concentrated in patients with a life expectancy sufficient to reach that point [5]. These studies already laid the groundwork for the structural nature of the benefit—real, but deferred—which the Arterial Revascularisation Trial (ART) would later replicate in the most rigorous form of randomised data.

At the same time, the technical aspect was developed. Two in situ arterial grafts, with their characteristic limited length, do not allow all territories to be reached; the solution was the composite graft, in which the right mammary artery, harvested as a free graft, is anastomosed to the left in a Y- or T-configuration to extend arterial revascularisation to the lateral and inferior walls without resorting to the aorta as a source of flow [6]. The extension of this concept to other arterial grafts—the radial, the inferior epigastric—in a composite configuration demonstrated better mid-term angiographic results compared with the free aorto-coronary graft, and established the rationale that still governs arterial assemblies today: the inflow is that of the in situ LIMA, and the patency of each branch depends on the demand of the perfused territory [7]. Large 15-year angiographic cohorts confirmed that the right mammary artery retains patency comparable to that of the left when anastomosed to an appropriate target, whilst the other arterial grafts share this longevity provided there is severe native stenosis [8].

The 2001 systematic review synthesised the available observational evidence and derived an estimate of the reduction in the risk of death that still serves as the benchmark [9], and over the following two decades, meta-analyses with adjusted hazard ratios and propensity-matched comparisons have replicated this finding with remarkable consistency [10,11]. The picture became more complicated in 2018, when a patient-level pooled analysis of randomised trials on the radial artery demonstrated a reduction in major cardiac events compared with the saphenous vein: randomised evidence in favour of a second arterial graft, but not of the right mammary artery in particular [12]. Since then, the question has no longer been whether the second graft should be arterial, but rather which artery, in what configuration and for which patient. It is this question, not the original one, that the decision-making model proposed here aims to answer.

## 3. A Real but Delayed Benefit

The superiority of the bilateral mammary artery approach over the single mammary artery approach in terms of long-term survival is documented by the seminal meta-analysis [9], with an HR for death of 0.81, and confirmed by updates based on large observational cohorts with adjusted estimates [10,11,13]. However, two caveats are crucial. Firstly, compared with single mammary artery bypass plus radial artery bypass, the advantage of bilateral mammary artery bypass only becomes apparent after more than ten years’ follow-up, not before [14,15]. Secondly, the randomised data have painted a more severe picture: at ten years, the ART showed no difference in the intention-to-treat analysis, whilst suggesting a benefit in the as-treated analyses and in high-volume centres [1,16,17]. It follows that the benefit must be earned: it requires a sufficient life expectancy and an appropriate technical setting. A caveat on the nature of this evidence is required before the argument proceeds. With the exception of the ART, the body of work supporting the superiority of the bilateral mammary artery consists of observational series and propensity-matched comparisons, in which matching removes measured imbalances but cannot remove the selection that led the surgeon to choose two mammary arteries in the first place; a formal analysis of unmeasured confounding in these studies has shown that a confounder of plausible magnitude would suffice to account for a substantial part of the apparent benefit [18]. The randomised comparison, for its part, remains the reference standard and has not confirmed the advantage on an intention-to-treat basis [16]. Throughout this article, accordingly, the superiority of the bilateral mammary artery is presented as a consistent and biologically plausible association rather than as an established causal effect, and the three-tier model is proposed as an instrument for identifying the patients in whom that association is most likely to translate into individual benefit.

## 4. Right Mammary or Radial? A Comparison with the Arterial Alternative

The most relevant comparison for current practice, however, is not with the single mammary plus veins approach, but with the single mammary plus radial approach, which the guidelines recommend as a Class I option for patients with severe stenosis and which represents the arterial alternative carrying the lowest risk of sternal complications [19]. The meta-analytic comparison of twelve matched cohorts—6450 patients with bilateral mammary bypass versus 9428 with mammary plus radial bypass—showed comparable survival rates over the first ten years (80.0% versus 79.9%) and a benefit of the bilateral mammary approach that only becomes apparent beyond this threshold, with an HR of 0.77 and a fifteen-year survival rate of 68% compared with 64 per cent [14]. The network meta-analysis of the three approaches confirms the hierarchy: the radial and right mammary arteries are both superior to the vein, and the difference between the two arteries is modest and time-dependent [15]. The most recent data, from a cohort of 2882 patients in Uruguay followed for two decades, adds an unexpected finding: the bilateral mammary approach was associated with lower operative mortality than the radial approach, with no differences in deep sternal infections or repeat revascularisations, whilst the long-term survival benefit (HR 0.73) did not withstand sensitivity analyses [20]. None of these findings argues against the bilateral mammary approach; taken together, they define its place: a graft whose advantage over the radial artery is long-term, and which should therefore be preferred when life expectancy justifies it and the geometry of the target site permits it—not as a matter of principle, but by selection. These comparisons, it must be added, are between matched cohorts rather than randomised groups: the hierarchy they establish is the most reliable currently available, but the individual point estimates are better read as ordering the options than as quantifying a causal effect—as the fragility of the Uruguayan hazard ratio under sensitivity analysis itself illustrates [18,20].

## 5. The Trade-Off Between Early Risk and Long-Term Benefit: A False Dilemma

A recurring question arises regarding the choice of the second conduit: since a failed revascularisation using both mammary arteries can be rectified, would it not be more prudent to use a single mammary artery plus veins, thereby avoiding the complexity of a branched composite graft? The question is legitimate but rests on a premise that the data do not support, namely that arterial complexity entails a cost in terms of early mortality.

## 6. Flow Competition as a Phenomenon of Patency, Not Mortality

Flow competition in a branched vessel—the mechanism underlying the ‘string sign’—is a phenomenon of patency, not acute mortality. When a branch supplies a territory where the native stenosis does not warrant it, the native flow remains predominant and the branch involutes over the following weeks, not in the operating theatre. The available data do not show an excess of perioperative myocardial infarctions in patients with flow competition, and early mortality in composite grafts stems not from the graft configuration but from the patient’s comorbidities. The risk to be balanced is therefore asymmetric compared to how it is framed: not early death from flow competition versus late survival, but late loss of patency if the target is poorly chosen versus late benefit if it is well chosen. Both outcomes are long-term and depend on the same variable: the appropriateness of the target.

## 7. Anastomotic Risk and the Primacy of Efficacy over Complexity

With regard to anastomotic risk, the evidence contradicts the cautious intuition. In a cohort of 3685 CABG procedures with a mean follow-up of thirteen years, early mortality was 1.6 per cent and was not influenced by the surgical technique—neither by the number of anastomoses, nor by the use of two mammary arteries, nor by the sequence or the creation of composite grafts [21]. On multivariate analysis, the number of distal arterial anastomoses proved to be an independent predictor of long-term survival, taking precedence over the number of grafts and the completeness of the revascularisation itself, with its discriminatory effect ceasing beyond three anastomoses [21]. Every correctly performed arterial anastomosis adds no measurable early risk and confers a long-term benefit: technical complexity, in itself, is not a risk factor.

The determinant of early risk is ineffective revascularisation, not complex revascularisation. In a series of 1000 patients with 98 per cent arterial grafts, operative mortality was 3.8 per cent overall, but 8.6 per cent in patients with incomplete revascularisation compared with 3.2 per cent in those with complete revascularisation [22]. Incompleteness more than doubles operative mortality. A single mammary artery graft plus a vein is therefore not a risk-free option: the vein carries its own risk of late failure, whilst a multiple arterial network mitigates the cost of any incompleteness where the vein exacerbates it; with arterial grafts, incompleteness is not associated with higher medium-term mortality in patients under eighty, whilst it is clearly associated with higher mortality in those over that age [22]. The contrast between the immediate safety of the single mammary plus vein approach and the risky long-term durability of the bilateral mammary approach is, to a large extent, a false dilemma: perioperative risk does not distinguish between the two strategies but between effective and ineffective revascularisation. The bilateral mammary bypass is no more risky; it is more demanding in terms of patient selection, and this requirement is rewarded with superior long-term survival whilst early-stage risk remains unchanged.

## 8. Anastomotic Errors and Intraoperative Monitoring

If the early risk lies not in complexity but in ineffectiveness, the term ‘ineffective’ must be broken down, as it encompasses two errors of differing severity: the strategic error—grafting a branch onto a target that does not warrant it—and the anastomotic error, namely anastomotic stenosis, torsion, an intimal flap or kinking.

## 9. A Hierarchy of Errors: Operative Strategy Versus Anastomotic Construction

The two errors are not equivalent. A strategic error leads to flow competition and involution of the graft, but the territory survives the lack of efficacy precisely because its native circulation was not so compromised—this, after all, was the cause of the competition. An anastomotic error, on the other hand, results in occlusion of a graft-dependent territory: native blood flow is cut off, leading to acute ischaemia within the first few hours, rather than silent thinning at a later stage. The clinical difference lies between patency that deteriorates over time and ischaemia that manifests in the recovery room. The two errors also differ in terms of correctability: a strategic error is difficult to rectify on the spot, whilst an anastomotic error is, in most cases, immediately rectifiable—provided it is identified. In mortality series involving low-risk patients, almost half of early deaths are preventable, and in the majority of cases the problem is graft thrombosis secondary to a technical error during harvesting or anastomosis [23].

## 10. Flow and Imaging: Intraoperative Monitoring as a Complement to Arterial Selection

It is precisely this class of error that intraoperative monitoring detects. Transit-time flow measurement identifies 2–4% of grafts requiring reassessment, with an impact on outcomes and not merely on parameters: in a cohort of 1000 arterial grafts, mortality in patients with a pulsatility index greater than 5 was 9% compared with 2%, and MACE was 17% compared with 5.4%, even when other intraoperative checks were reassuring [23]. That the measurement may do more than predict—that acting upon it may alter the course of events—is suggested, though not established, by the propensity-matched analysis of 910 patients undergoing total arterial revascularisation, in which MACE rates were 3.3 per cent with flow measurement versus 6.9 per cent without, showing an independent protective effect despite longer cardiopulmonary bypass times [24]. Here too the design sets the limit of what may be concluded: the centres and the operators who adopt flow measurement are unlikely to differ from those who do not in that respect alone, and propensity matching cannot adjust for a disposition towards verification that is itself unmeasured. The argument for intraoperative verification developed below therefore rests on the consistency of the mechanism—an error detected is an error that can be corrected—rather than on the magnitude of these adjusted estimates.

Flow measurement assesses function but does not visualise the anastomosis; imaging does visualise it. High-resolution epicardial ultrasound overcomes the main limitation of transit-time flowmetry—its low positive predictive value—by identifying, amongst grafts deemed functional based on flow measurements, a proportion of grafts that are in fact malfunctioning (later confirmed as technical errors upon review), thereby bringing diagnostic accuracy close to 100 per cent [25,26]. Flow measurement and imaging are complementary rather than alternative: the former indicates whether blood is passing through, the latter what the cross-section of the passage looks like; a combination that is all the more useful in bilateral mammary bypass grafts, where the vessels have low flow and high competitive sensitivity and where interpreting flow alone is more difficult.

The crucial question remains: based on what has been published from 2010 to the present, can it be considered unjustifiable not to carry out such checks, especially given that they are available in the operating theatre? The 2018 ESC/EACTS guidelines assign routine flow measurement a Class IIa recommendation, Level of Evidence B (“should be considered”) rather than Class I, acknowledging themselves that interpretation is challenging in sequential and T-shaped configurations [19]. In a strictly regulatory sense, therefore, omitting it does not constitute a deviation from the standard. The strongest argument, however, is not regulatory but relates to the internal consistency of the surgical choice [27,28]. Those who opt for the more demanding strategy—composite bilateral mammary bypass, with multiple Y- or T-shaped anastomoses, the long-term benefit of which depends entirely on the patency of fragile conduits—and forgo intraoperative monitoring accept the complexity that generates the benefit whilst rejecting the tool that protects against what that complexity might cause to fail. When one adds the possible presence of the equipment in the operating theatre, the pragmatic justification for its non-use also falls away. The correct formulation is not that the guidelines mandate it, but that those who choose arterial complexity and have the equipment available have no rational justification for omitting it: intraoperative monitoring is the logical corollary of the decision to revascularise using two arteries.

## 11. First Level: Absolute Contraindications

Before assessing the risk, it is necessary to rule out any medical history that precludes the use of the conduit from the outset. Unlike risk factors that can be assessed, these cannot be mitigated: they must be adhered to.

### 11.1. Damage to the Conduit Upstream: Thoracic Radiotherapy

Previous thoracic radiotherapy—typically for Hodgkin’s disease, but also following mastectomy or for mediastinal neoplasms—compromises the integrity of the mammary conduit because it causes wall fragility and mediastinal fibrosis, leading to intraoperative damage and poor sternal healing, rather than through radiation-induced atherosclerosis [29,30,31]. The vessel may appear angiographically intact yet still fail, and in irradiated patients the excellent long-term patency observed in the general population is not guaranteed [32]. The long-term survivor of Hodgkin’s lymphoma is the clinical archetype of this scenario.

On this point, the literature warrants a distinction, because the significant damage is not what one might expect. Mediastinal irradiation accelerates coronary atherosclerosis in a dose-dependent manner, with no apparent threshold [31,33], but the internal mammary artery appears to be largely spared from this process. The most extensive histomorphological study available—133 irradiated mammary segments compared with 133 non-irradiated controls—identified neither severe fibrosis nor radiation-induced damage to the conduit, with the lumen remaining largely patent and no differences between the irradiated and contralateral sides [29], and the corresponding clinical series show no excess incidence of graft failure or mortality attributable to the use of one or both mammary arteries in irradiated patients [29]. On this basis, reviews on post-radiation cardiopathy recommend the use of the mammary artery whenever feasible, whilst making this contingent upon a thorough assessment of the vessel [34,35]. This finding is confirmed radiographically: in a cohort of irradiated patients studied by CT and mammography several years later, the mammary artery showed no signs of radiation-induced atherosclerosis, and calcification of its branches was independent of the irradiated side [36].

This leads to a clarification that refines, without overturning, the logic of the first level. What makes the irradiated mammary artery a treacherous vessel is not an intrinsic disease of the arterial wall—which is angiographically predictable—but the combination of friability at harvesting and fibrosis of the mediastinal bed, which results in intraoperative damage and precarious sternal healing rather than stenosis of the vessel itself [29,30]. This is why the conduit may appear unharmed yet still fail—and why angiographic assessment of the conduit prior to use is prudent rather than superfluous [34,35]—and why the contraindication, where it applies, rests on the risk to healing and the integrity of the wall rather than on the primacy of radiation-induced atherosclerosis. Thoracic radiotherapy therefore remains a contraindication to be observed, but its significance must be assessed on a case-by-case basis—taking into account dose, time elapsed and the quality of the sternal bed—rather than being treated as an absolute and uniform prohibition.

### 11.2. The Haemodynamically Trapped Conduit: Subclavian Pathology

A subclavian stenosis proximal to the origin of the LIMA reverses the pressure gradient, generating retrograde flow from the graft towards the subclavian artery at the expense of myocardial perfusion: coronary-subclavian steal [37]. Subclavian stenosis affects approximately 2% of the general population, with a higher prevalence in vascular patients [38], and must be ruled out using dedicated imaging when clinically suspected. This represents the case of a healthy conduit that is poorly connected upstream, and forms the conceptual bridge to the second level.

### 11.3. The Conduit Without Reliable Inflow: Acute Aortic Dissection

In acute type A aortic dissection, the priority is aortic repair and the management of coronary and supra-aortic trunk hypoperfusion, which complicates a significant proportion of cases [39]. In this context, the mammary artery does not provide a reliable inflow, and revascularisation, when indicated, is performed concurrently with the repair using different grafts. The evidence specifically addressing this point is indirect, but the pathophysiological rationale is consistent with the literature on hypoperfusion in this scenario.

The principle common to the three first-level scenarios is that the mammary artery is only as good as its origin. A biologically excellent graft with compromised inflow—irradiated upstream, trapped by a subclavian stenosis, or dependent on a dissected aortic arch—is an unreliable graft, and no technical skill in distal anastomosis can compensate for the upstream defect. This is why these three conditions logically and chronologically precede any assessment of the target and sternal risk: one cannot grade what cannot be used.

## 12. Second Level: The Conduit and Haemodynamic Alignment

### 12.1. The Target: Critical Stenosis and Flow Demand

Once the filter of absolute contraindications has been passed, the nature of the conduit as a living structure with finite flow reserve remains. The mammary artery adapts its calibre to the demand of the perfused territory: when the native stenosis is not severe, or when an adjacent branch provides competitive flow, the graft undergoes involution. It is worth stating what ‘not severe’ means in numerical terms, since the threshold governs the decision. A diameter stenosis of at least 70% should be regarded as the minimum requirement for any arterial branch, and one of 80% or more as preferable for the branches of a composite graft, in which a single inflow is shared among several territories and the least diseased target dictates the behaviour of the whole assembly; the recommendation that the second arterial conduit be directed towards vessels with severe proximal stenosis derives from precisely this consideration [19]. Below these values competitive flow becomes the dominant haemodynamic condition and the probability of graft involution rises steeply, so that a target of intermediate severity is better served by a venous graft—whose larger calibre and lower resistance tolerate competition—or left ungrafted altogether where the territory at stake is small [40,41]. The phenomenon known as ‘string sign’ is systematically associated with competitive flow as the basis for graft involution [40]. In composite bilateral mammary graft assemblies, competitive flow may reverse into coronary-to-coronary shunting with retrograde flow along the graft [6,41,42,43]. It is this mechanism that underlies the risk of early malperfusion, a potential determinant of haemodynamic instability and perioperative mortality. This gives rise to three operational imperatives: selecting a target with critical stenosis and high blood flow demand, choosing a suitable graft configuration, and validating blood flow intraoperatively before concluding the procedure.

### 12.2. The Configuration: In Situ, Y- or T-Shaped Composite, or Free Graft

The second level concerns not only the choice of target, but also the geometry by which the conduit reaches it. The right mammary artery may be used in situ, either through the transverse sinus towards the circumflex artery or anteriorly towards the LAD; as a free aorto-coronary graft; or as a branch of a composite graft on the left mammary artery. Each configuration has its own flow profile. The in situ graft benefits from direct pressure from the subclavian artery and tolerates competition better; the free graft from the aorta provides a full inflow but exposes the graft to a proximal anastomosis on the aortic wall, which may compromise its patency; and the composite configuration makes the entire revascularisation dependent on a single inflow—the LIMA—and distributes a finite flow amongst multiple branches, with a sensitivity to competition that increases with the number of anastomoses and the degree of stenosis in the least diseased target vessel [6,40,42]. Alongside the flow profile, each configuration carries a constraint of anatomical reach, which in practice often settles the choice. The in situ right mammary artery, routed through the transverse sinus, reaches the proximal circumflex and its first marginal branch reliably, but it frequently falls short of the distal circumflex and of the posterolateral and inferior territories—the more so in the dilated heart, in the tall patient, and whenever the vessel has been harvested as a pedicle, which costs useful length. Where reach is the limiting factor, the composite Y-graft is the solution: the right mammary artery, divided and anastomosed to the left trunk, is relieved of its subclavian origin and gains the length required to reach targets that no in situ route can serve, which was the original rationale for the composite conduit [6]. Skeletonized harvesting works in the same direction, adding both length and free flow, and the two expedients are commonly combined. The practical rule is that the configuration should be chosen once the targets have been identified, and not before: it is the topography of the vessels to be grafted that determines whether the right mammary artery is better used in situ or as a branch, rather than a general preference for one assembly over the other.

Clinical data support the composite configuration when it is entirely arterial. A comparison between two institutions—one favouring a bilateral mammary approach with additional non-sequential veins (436 patients) and the other favouring a sequential arterial Y-graft for all target vessels (771 patients)—with an equal average number of anastomoses (four per patient) and no differences in in-hospital events, showed superior long-term survival in the Y-graft group; the same authors acknowledge, however, that the rate of competitive flow in the composite configuration is higher than in the in situ configuration, and that the function of the right branch depends on the severity of the stenosis in the circumflex branch or the right system it serves [41]. Similarly, revascularisation using only a bilateral mammary artery in a T-configuration, in 2098 patients compared with 1659 revascularised with a single mammary artery plus other grafts, resulted in a ten-year survival rate of 81% versus 72% in the propensity-matched pairs, with comparable in-hospital mortality (1.6% versus 2.9%) [43]. The composite graft, therefore, is not a fallback option compared with the in situ configuration; it is a strategy with its own body of evidence, provided that target selection and intraoperative flow verification guide its implementation.

The configuration interacts with the first level in a non-obvious way. The Y- or T-shaped composite shifts the origin of the right mammary artery from the right subclavian to the left mammary artery: in the presence of left subclavian stenosis, the entire arterial revascularisation becomes dependent on a compromised inflow, and coronary-subclavian steal extends to all the territories supplied; conversely, in the presence of right subclavian stenosis, converting the right mammary artery into a composite branch is the solution, not the problem [37,38]. This is a concrete example of how the three levels do not operate independently of one another: the geometry chosen at the second level alters the weighting of the contraindication assessed at the first, and the intraoperative assessment of flow—which, in the composite approach, measures the entire system via the common trunk of the LIMA—becomes the tool that brings the process full circle.

## 13. Third Level: The Patient and Sternal Risk

### 13.1. Diabetes, Skeletonization and Residual Sternal Risk

The final level is where the decision becomes probabilistic. Sternal healing is the bottleneck of the bilateral mammary procedure, and diabetes has historically been the main determining factor. In the classic series, bilateral mammary artery harvesting increased the incidence of deep sternal infection from 1.3 per cent to 14.3 per cent in diabetic patients, to the extent that it was considered a contraindication [44]. The situation was reversed by skeletonization: by preserving sternal vascularisation, it has reduced the risk of infection to such an extent that diabetes is no longer a contraindication, provided that both mammary arteries are harvested in a skeletonized fashion [45,46]. Meta-analyses confirm that the higher incidence of deep sternal infections associated with bilateral mammary artery grafting—approximately 3.3 per cent versus 1.7 per cent—disappears with the skeletonized technique [47].

This does not negate the patient’s weight. In cohorts with strict glycaemic control and predominantly off-pump surgery, the incidence of deep sternal infection is similar between single and bilateral mammary artery grafting, but diabetes remains a residual risk factor despite skeletonization [47,48]. Obesity, chronic obstructive pulmonary disease, immunosuppression and the urgency of the procedure all contribute to defining the risk profile. The patient modulates the risk–benefit ratio; the technique mitigates it but does not eliminate it entirely.

### 13.2. Beyond Diabetes: Ventricular Dysfunction, Renal Failure, Advanced Age

The third level is not limited to sternal risk. The patient also modulates the risk–benefit ratio in terms of life expectancy and functional reserve, which determine whether the deferred benefit will have time to materialise. Three subpopulations are instructive, as they have long been excluded from bilateral mammary artery grafting due to an excess of caution not justified by the data.

In left ventricular dysfunction, the evidence is nuanced. In 4537 patients stratified by ejection fraction and matched using propensity scores, bilateral mammary bypass did not increase operative mortality in any group—including that with an ejection fraction below 30 per cent, where it was 6.9 per cent compared with 10.3 per cent for single bypass—nor did it increase sternal complications, and it improved ten- and twenty-year survival in the groups with intermediate (33% versus 19% at twenty years) and preserved function, whilst in the most severely compromised group the benefit did not reach statistical significance due to the limited expected survival [49]. A more recent study involving patients with an ejection fraction of 40% or less, matched across 111 pairs, found no survival benefit at fifteen years, identifying insulin-dependent diabetes, perioperative counterpulsation and deep sternal infection as independent predictors of late mortality—the latter with an HR greater than 7 [50]. A Japanese multicentre experience subsequently reported a survival benefit even in cases of severe dysfunction associated with diabetes [51]. A consistent interpretation of these data is not that bilateral mammary artery grafting is contraindicated in patients with compromised ventricular function, but that their benefit is contingent upon a life expectancy which is reduced by the dysfunction itself, and that the cost of a sternal complication is, in these patients, particularly high: skeletonization is not an option here; it is a prerequisite.

In renal failure, historically considered a relative contraindication due to the risk of infection, recent data suggest the opposite. In 391 patients with a glomerular filtration rate below 60 mL/min/1.73 m^2^, skeletonized bilateral mammary bypass did not increase 30-day mortality (5.6% versus 9.0%) nor sternal infections (2.2% versus 5.7%), and was associated with double the median survival—8.4 versus 4.1 years—with an independent reduction in late mortality even in the matched cohort [52]. The authors put forward a hypothesis that merits attention: in patients with atherosclerosis accelerated by uraemia, venous vessel degeneration occurs more rapidly, and the advantage of using an artery becomes apparent at an earlier stage than in the general population. In other words, the high-risk patient may be precisely the one who can least afford to rely on a venous graft.

Finally, in the elderly, the criterion of life expectancy should be applied with caution. In 1300 patients aged 70 or over, the bilateral mammary bypass in a T-shaped configuration yielded a 10-year survival rate of 66 per cent compared with 53 per cent for the single mammary bypass in matched pairs, with an HR of 0.66 and comparable post-operative morbidity [53]. The age limit beyond which the benefit is lost is therefore not fixed and depends less on chronological age than on expected survival, net of comorbidities. The practical rule is that a patient who, at the age of seventy, can reasonably be expected to have ten years left to live is still a suitable candidate, provided that sternal risk is contained by the technique used and that the patient’s frailty does not compromise their recovery.

## 14. The Cross-Cutting Variable: Harvesting Technique and Learning Curve

The three levels described so far presuppose technically flawless execution, a premise that must be made explicit. A significant proportion of the risk historically attributed to bilateral mammary artery grafting as such is, in reality, the risk of imperfect harvesting and an incomplete learning curve. The harvesting technique and the surgeon’s experience do not constitute a fourth parallel level, but rather a confounding variable that cuts across and re-interprets the three preceding levels.

### 14.1. Harvesting Techniques: Pedicled, Skeletonized, Semi-Skeletonized

Pedicled harvesting removes the artery en bloc with the satellite veins, adipose tissue and endothoracic fascia; skeletonization isolates the vessel alone, stripping it of perivascular tissues [54]. Skeletonization preserves sternal vascularisation—retrosternal microcirculation is significantly less compromised compared with the pedicled method in randomised laser-Doppler studies [55]—provides a longer conduit, greater free flow and allows for more sequential anastomoses. This preservation explains the key clinical finding: in the ART sub-study, the pedicled bilateral mammary graft increased the risk of sternal complications (OR 1.80), whereas the skeletonized bilateral mammary graft did not (OR 1.00) [56]. Meta-analyses confirm the reduced risk of sternal infection with the skeletonized technique (OR approximately 0.45) [57]. The semi-skeletonized variant, which spares one satellite vein, represents a useful compromise during a technical transition phase.

### 14.2. The Pitfalls of Skeletonization

Skeletonization is a more demanding and slower harvesting technique, and the surgeon’s manoeuvres close to the vessel wall entail specific risks: intimal dissection, subadventitial haematoma, thrombosis and spasm [58]. Endothelial damage is the key mechanism: the arterial endothelium secretes potent vasodilators, and even minimal injury (direct grasping of the intima, excessive traction, high-power electrosurgical units) can trigger spasm, described as the Achilles’ heel of arterial conduits [58]. This has led to established operating guidelines: avoid direct grasping of the artery, use low-power currents (10–20 W), apply topical spasmolytics (papaverine) and employ hydrodissection to create the working plane whilst minimising heat transfer [59]. Kinking of the skeletonized conduit and, above all, twisting—given its greater length and mobility—must be carefully prevented by monitoring the course of the graft, planning the topography of the anastomoses and, where possible, securing its course with delicate adventitial sutures (8/0), taking care not to create anchor points that might themselves cause constrictions or angulations. This level of care is particularly important in sequential revascularisation or when using the T- or Y-shaped composite graft technique. The intraoperative distinction between spasm and intimal dissection in a low-flow graft represents one of the most delicate and experience-dependent decision-making moments: inappropriate endoluminal dilation on an unrecognised dissection is a potentially catastrophic error.

### 14.3. The Learning Curve and Its Confounding Effect

The bilateral mammary bypass is a longer and more complex operation, associated with a genuine learning curve. CUSUM analyses in coronary surgery indicate that, with regard to LIMA harvesting and coronary bypass grafting, the surgeon achieves autonomy after a few dozen cases [60], but the transition to the skeletonized bilateral mammary bypass raises the threshold: the literature defines an operator as ‘expert’ once they have performed more than 50 procedures and explicitly examines a learning curve effect by excluding a surgeon’s earliest cases [61]. In minimally invasive and robotic contexts, proficiency is attained after several hundred cases [62]. This calls for interpretative caution: the unfavourable signals regarding skeletonization—lower patency and a higher risk of events in the post hoc analyses of COMPASS and ART, and higher long-term mortality in a Swedish nationwide registry [61,63,64]—are difficult to separate from the surgeon’s experience and the centre’s case volume in observational datasets. Consistent with this, in the ART study, the advantage of the bilateral mammary approach emerged only in the analysis restricted to high-volume surgeons [16]. Before attributing an unfavourable outcome to the conduit, it is therefore necessary to rule out the possibility that it is attributable to the surgeon.

## 15. Missing Data and Revealing Data: Hidden Mortality and Utilisation Rates

### 15.1. The Hidden Lesion as an Inferred Phenomenon

One question deserves an explicit answer: what is the perioperative mortality attributable to malperfusion resulting from an occult injury to the conduit during harvesting? As a discrete category, this data does not exist, and there is a methodological explanation for this absence: a lesion not recognised intraoperatively is not coded as an independent cause, but is subsumed within broader categories (perioperative infarction, low-output syndrome, early graft failure). No register has a field for death due to occult damage to the mammary artery.

The available evidence addresses the phenomenon without isolating it. Even in the best-characterised setting—injury to a patent mammary graft at coronary reoperation—recognised injury has a documented prevalence of around 5.3 per cent and, when revascularisation of the LAD remains ineffective, is associated with perioperative infarction and operative mortality of the order of 8.6 per cent [65]. Early graft failure manifesting as perioperative myocardial infarction has an overall incidence of between 2% and 10%, rising to 30% depending on the definition, with a deterioration in short- and long-term survival [66]. In none of these datasets can the occult component be distinguished from other causes.

This apparent gap, however, turns into an argument. The occult lesion, which cannot be measured retrospectively, can be detected at the very moment it occurs: this is the purpose of transit-time flow measurement (TTFM). Arterial grafts are the vessels most susceptible to harvesting injury and competitive flow, and therefore those that benefit most from intraoperative assessment [67]. The thresholds are clear and well-defined: Mean Graft Flow (MGF) not exceeding 15–20 mL/min, a pulsatility index (PI) of 5 or higher, and elevated backward flow (%BF) [68]. In a series of 910 patients undergoing total arterial revascularisation, TTFM reduced MACE from 6.9% to 3.3% [24]. Mortality from occult lesions is therefore not a quantifiable figure, but rather a phenomenon whose existence is inferred from the very efficacy of flow assessment: it is the absence of this data that justifies systematic intraoperative validation, not that it renders it superfluous.

### 15.2. The Impact of Data Publication on Usage Rates

Whether the publication of data has altered the use of bilateral mammary bypass over time is a question for which there is documented evidence, although the link is not derived from malperfusion data (which do not exist as a discrete category) but from outcome data. In the United States, usage has remained chronically low and essentially stable: 3.5 per cent in 1999 and 4.1 per cent in 2009 out of approximately 1.5 million patients, i.e., almost no increase over a decade [69]. Following a modest peak around 2015–2016, a gradual decline was observed, becoming more pronounced after 2016 and again after 2019 [70]. These two dates correspond to the publication of the 5-year (December 2016) and 10-year (January 2019) results of the ART trial, both of which were neutral on the primary endpoint. The literature on the barriers to multiple arterial revascularisation is clear: many surgeons have drawn their own conclusions from the neutral trials, and the burden of sternal complications—sternal reconstructions occurring three times as frequently, 1.9 per cent versus 0.6 per cent—has outweighed the deferred benefit [17,70].

Added to this is the economic dimension, which is rarely explicitly addressed in the surgical debate. The cost-effectiveness analysis conducted on the ten-year ART data, from the perspective of the UK National Health Service, found an average cost that was approximately £1100 higher in the bilateral mammary artery group, with no difference in quality-adjusted life years, and a probability of cost-effectiveness of 33 per cent at the conventional threshold of £20,000 per QALY; the result only became favourable in extrapolations beyond the trial’s time horizon [71]. The economic data, therefore, also reiterates the clinical lesson set out in Section 3: bilateral mammary artery is a long-term investment and, as such, should be offered to those who have the time to reap its benefits, which reinforces, rather than weakens, the need for structured selection.

This gives rise to a paradox that brings the discussion full circle to its starting point: the publication of high-quality randomised evidence has discouraged the adoption of a technique that observational meta-analyses continue to show is beneficial: the documented discrepancy between randomised and observational evidence [18,72]. Bilateral mammary artery use thus represents an almost unique case in which the pinnacle of the evidence pyramid has reduced, rather than increased, the use of a procedure: a further reason not to base the decision on a single aggregated figure, but on the structured hierarchy proposed here. One final reading of the utilisation figure deserves to be made explicit, because it is the opposite of the one usually offered. A rate of 5 to 10 per cent is routinely presented as a therapeutic shortfall, and clinical inertia is the inference drawn from it; however, the three-tier sequence set out here, applied rigorously, excludes a considerable proportion of the patients coming to coronary surgery—those in whom a contraindication applies, those whose second target does not warrant an arterial branch, those whose sternal risk or life expectancy does not repay a deferred benefit, and those operated upon by a surgeon who has not completed the relevant learning curve. The appropriate rate of bilateral mammary artery use is, in other words, not 100 per cent, and is unlikely to be a figure that can be stated in the abstract; a low observed rate is as consistent with disciplined selection as with undue conservatism, and the two cannot be told apart from the aggregate figure alone. What would tell them apart is the reason recorded for each patient in whom the second mammary artery was not used: the question that matters is not how many patients receive two mammary arteries but whether those who do not receive them are denied them for a reason belonging to one of the three levels. On this, registries are silent, and the deficiency is one of documentation rather than of surgical judgement. The decline observed after 2016 and 2019 is thus open to a less pessimistic interpretation than the one usually placed upon it: in part it reflects surgeons incorporating neutral randomised results into their practice, which is precisely what the hierarchy of evidence asks of them, and only in part a retreat from a technique whose benefit is real but conditional.

## 16. Conclusions

The 2024 European guidelines confirm the preferential role of coronary artery bypass grafting, with a strong recommendation for diabetic patients, where surgical revascularisation is recommended over both percutaneous procedures and optimal medical therapy alone [73]. In this context, the choice of multiple arterial grafts becomes central. The underutilisation of the bilateral mammary approach cannot be rectified by indiscriminate enthusiasm, but through a structured three-tiered selection process: excluding the procedure when an absolute contraindication precludes it; aligning the graft with the haemodynamics of the target territory; and grading sternal risk in the individual patient. All this must be supported by an appropriate harvesting technique and a fully mastered learning curve; without these, every level of decision-making loses its reliability. It is neither a one-size-fits-all technique, nor one to be abandoned, but a technique to be applied with precision and performed with experience: a delayed benefit that warrants a structured decision.

In practical terms, the sequence is simple to set out but more difficult to follow. At the first level, one asks whether there is any reason in the patient’s medical history why the conduit cannot be used: thoracic radiotherapy (to be assessed on a case-by-case basis, not automatically ruled out), subclavian artery stenosis, acute aortic dissection. At the second level, one considers whether the target warrants the right mammary artery—critical stenosis, high-demand territory—and in what geometry to access it, bearing in mind that the composite configuration shifts the origin of the conduit and redefines its dependencies. Thirdly, one must consider whether the patient has time to reap the benefits and how much a sternal complication would cost in that particular patient, and the answer is all the more favourable the more thoroughly skeletonization has been mastered. Running through all three levels is the question regarding the surgeon, which is the least comfortable but the most decisive: a bilateral mammary procedure performed by someone who has not completed their learning curve is not the same operation whose benefits are documented in the literature. Closing the sequence is the intraoperative monitoring of flow and imaging, which is not an additional level but rather the verification that the previous three have been correctly adhered to.

Two qualifications belong in this conclusion rather than in a footnote. The first concerns the strength of the claim. The advantage of the bilateral mammary artery over the arterial alternatives is supported mainly by observational and propensity-matched evidence, is modest and late in its appearance, and has not been confirmed on an intention-to-treat basis by randomisation [16,18,72]; what is proposed here is accordingly a method of selection and not a verdict of superiority, and it would retain its usefulness even were the effect to prove smaller than the observational literature suggests. The second concerns the word underutilisation, which should be used with care. The proportion of patients who ought to receive two mammary arteries is itself determined by the three levels, and a low rate in a given centre is no evidence of a shortcoming unless the patients excluded were excluded for reasons that belong to none of them. The purpose of a decision framework is not to raise the rate, but to ensure that whatever rate results is the product of selection rather than of habit.

## Data Availability

No new data were created or analysed in this study. Data sharing is not applicable to this article.

## References

[1] Gaudino M., Bakaeen F., Benedetto U., Rahouma M., Di Franco A., Tam D.Y., Iannaccone M., Schwann T.A., Habib R., Ruel M. (2018). Use rate and outcome in bilateral internal thoracic artery grafting: Insights from a systematic review and meta-analysis. J. Am. Heart Assoc..

[2] Taggart D.P. (2019). Implications of the 10-year outcomes of the Arterial Revascularization Trial (ART) for multiple arterial grafts during coronary artery bypass graft. Eur. J. Cardiothorac. Surg..

[3] Loop F.D., Lytle B.W., Cosgrove D.M., Stewart R.W., Goormastic M., Williams G.W., Golding L.A., Gill C.C., Taylor P.C., Sheldon W.C. (1986). Influence of the internal-mammary-artery graft on 10-year survival and other cardiac events. N. Engl. J. Med..

[4] Lytle B.W., Blackstone E.H., Loop F.D., Houghtaling P.L., Arnold J.H., Akhrass R., McCarthy P.M., Cosgrove D.M. (1999). Two internal thoracic artery grafts are better than one. J. Thorac. Cardiovasc. Surg..

[5] Lytle B.W., Blackstone E.H., Sabik J.F., Houghtaling P., Loop F.D., Cosgrove D.M. (2004). The effect of bilateral internal thoracic artery grafting on survival during 20 postoperative years. Ann. Thorac. Surg..

[6] Calafiore A.M., Di Giammarco G., Luciani N., Maddestra N., Di Nardo E., Angelini R. (1994). Composite arterial conduits for a wider arterial myocardial revascularization. Ann. Thorac. Surg..

[7] Calafiore A.M., Di Giammarco G., Teodori G., D’Annunzio E., Vitolla G., Fino C., Maddestra N. (1995). Radial artery and inferior epigastric artery in composite grafts: Improved midterm angiographic results. Ann. Thorac. Surg..

[8] Tatoulis J., Buxton B.F., Fuller J.A. (2004). Patencies of 2127 arterial to coronary conduits over 15 years. Ann. Thorac. Surg..

[9] Taggart D.P., D’Amico R., Altman D.G. (2001). Effect of arterial revascularisation on survival: A systematic review of studies comparing bilateral and single internal mammary arteries. Lancet.

[10] Takagi H., Goto S.N., Watanabe T., Mizuno Y., Kawai N., Umemoto T. (2014). A meta-analysis of adjusted hazard ratios from 20 observational studies of bilateral versus single internal thoracic artery coronary artery bypass grafting. J. Thorac. Cardiovasc. Surg..

[11] Buttar S.N., Yan T.D., Taggart D.P., Tian D.H. (2017). Long-term and short-term outcomes of using bilateral internal mammary artery grafting versus left internal mammary artery grafting: A meta-analysis. Heart.

[12] Gaudino M., Benedetto U., Fremes S., Biondi-Zoccai G., Sedrakyan A., Puskas J.D., Angelini G.D., Buxton B., Frati G., Hare D.L. (2018). Radial-artery or saphenous-vein grafts in coronary-artery bypass surgery. N. Engl. J. Med..

[13] Urso S., Nogales E., González J.M., Sadaba R., Tena M.Á., Bellot R., Ríos L., Portela F. (2019). Bilateral internal thoracic artery versus single internal thoracic artery: A meta-analysis of propensity score-matched observational studies. Interact. Cardiovasc. Thorac. Surg..

[14] Urso S., Sadaba R., González-Martín J.M., Nogales E., Tena M.Á., Portela F. (2024). Bilateral internal thoracic artery versus single internal thoracic artery plus radial artery: A double meta-analytic approach. J. Thorac. Cardiovasc. Surg..

[15] Gaudino M., Lorusso R., Rahouma M., Abouarab A., Tam D.Y., Spadaccio C., Saint-Hilary G., Leonard J., Iannaccone M., D’Ascenzo F. (2019). Radial artery versus right internal thoracic artery versus saphenous vein as the second conduit for coronary artery bypass surgery: A network meta-analysis of clinical outcomes. J. Am. Heart Assoc..

[16] Taggart D.P., Benedetto U., Gerry S., Altman D.G., Gray A.M., Lees B., Gaudino M., Zamvar V., Bochenek A., Buxton B. (2019). ART Investigators. Bilateral versus single internal-thoracic-artery grafts at 10 years. N. Engl. J. Med..

[17] Taggart D.P., Altman D.G., Gray A.M., Lees B., Gerry S., Benedetto U., Flather M., on behalf of the ART Investigators (2016). Randomized trial of bilateral versus single internal-thoracic-artery grafts. N. Engl. J. Med..

[18] Gaudino M., Di Franco A., Rahouma M., Tam D.Y., Iannaccone M., Deb S., D’Ascenzo F., Abouarab A.A., Girardi L.N., Taggart D.P. (2018). Unmeasured confounders in observational studies comparing bilateral versus single internal thoracic artery for coronary artery bypass grafting: A meta-analysis. J. Am. Heart Assoc..

[19] Sousa-Uva M., Neumann F.-J., Ahlsson A., Alfonso F., Banning A.P., Benedetto U., A Byrne R., Collet J.-P., Falk V., Head S.J. (2019). 2018 ESC/EACTS Guidelines on myocardial revascularization. Eur. J. Cardiothorac. Surg..

[20] Montero J.A., Cubas S., Hernandez M., Sosa C., Soca G., Dayan V. (2026). Early and long-term outcomes of bilateral internal mammary artery versus radial artery grafting: A national cohort study. JTCVS Open.

[21] Jegaden O.J.L., Farhat F., Jegaden M.P.O., Hassan A.O., Lapeze J., Eker A. (2021). How decisive is the number of distal arterial anastomoses in coronary bypass surgery?. J. Cardiothorac. Surg..

[22] Kieser T.M., Curran H.J., Rose M.S., Norris C.M., Graham M.M. (2014). Arterial grafts balance survival between incomplete and complete revascularization: A series of 1000 consecutive coronary artery bypass graft patients with 98% arterial grafts. J. Thorac. Cardiovasc. Surg..

[23] Kieser T.M., Rose S., Kowalewski R., Belenkie I. (2010). Transit-time flow predicts outcomes in coronary artery bypass graft patients: A series of 1000 consecutive arterial grafts. Eur. J. Cardiothorac. Surg..

[24] Laali M., Nardone N., Demondion P., D’ALessandro C., Guedeney P., Barreda E., Lebreton G., Leprince P. (2022). Impact of transit-time flow measurement on early postoperative outcomes in total arterial coronary revascularization with internal thoracic arteries: A propensity score analysis on 910 patients. Interact. Cardiovasc. Thorac. Surg..

[25] Di Giammarco G., Pano M., Cirmeni S., Pelini P., Vitolla G., Di Mauro M. (2006). Predictive value of intraoperative transit-time flow measurement for short-term graft patency in coronary surgery. J. Thorac. Cardiovasc. Surg..

[26] Di Giammarco G., Canosa C., Foschi M., Rabozzi R., Marinelli D., Masuyama S., Ibrahim B.M., Ranalletta R.A., Penco M., Di Mauro M. (2014). Intraoperative graft verification in coronary surgery: Increased diagnostic accuracy adding high-resolution epicardial ultrasonography to transit-time flow measurement. Eur. J. Cardiothorac. Surg..

[27] Gaudino M., Sandner S., Di Giammarco G., Di Franco A., Arai H., Asai T., Bakaeen F., Doenst T., Fremes S.E., Glineur D. (2021). The use of intraoperative transit time flow measurement for coronary artery bypass surgery: Systematic review of the evidence and expert opinion statements. Circulation.

[28] Taggart D.P., Thuijs D.J., Di Giammarco G., Puskas J.D., Wendt D., Trachiotis G.D., Kieser T.M., Kappetein A.P., Head S.J. (2020). Intraoperative transit-time flow measurement and high-frequency ultrasound assessment in coronary artery bypass grafting (REQUEST). J. Thorac. Cardiovasc. Surg..

[29] Gansera B., Schmidtler F., Weingartner J., Angelis I., Kiask T., Kemkes B.M. (2007). Quality of internal thoracic artery grafts after mediastinal irradiation. Ann. Thorac. Surg..

[30] Brown M.L., Schaff H.V., Sundt T.M. (2008). Conduit choice for coronary artery bypass grafting after mediastinal radiation. J. Thorac. Cardiovasc. Surg..

[31] Jaworski C., Mariani J.A., Wheeler G., Kaye D.M. (2013). Cardiac complications of thoracic irradiation. J. Am. Coll. Cardiol..

[32] Wu W., Masri A., Popovic Z.B., Smedira N.G., Lytle B.W., Marwick T.H., Griffin B.P., Desai M.Y. (2013). Long-term survival of patients with radiation heart disease undergoing cardiac surgery: A cohort study. Circulation.

[33] Darby S.C., Ewertz M., McGale P., Bennet A.M., Blom-Goldman U., Brønnum D., Correa C., Cutter D., Gagliardi G., Gigante B. (2013). Risk of ischemic heart disease in women after radiotherapy for breast cancer. N. Engl. J. Med..

[34] Tang D., Peng E.W.K., Giri D., Chowdhary M., Sarkar P. (2009). Mediastinal irradiation and its effect on the cardiovascular system. Br. J. Hosp. Med..

[35] van Rijswijk S., Huijbregts M.A.J.M., Lust E., Strack van Schijndel R.J.M. (2008). Mini-review on cardiac complications after mediastinal irradiation for Hodgkin lymphoma. Neth. J. Med..

[36] Naraparaju V., Mosleh W., Almnajam M., Khaddr M., Bagade S., Posteraro A., Grew D., Wakefield D.B., Hur D.J., Vashist A. (2022). The utility of radiographic assessment of the internal mammary arteries in chest wall irradiated patients. Front. Biosci. (Landmark Ed.).

[37] Takach T.J., Reul G.J., Cooley D.A., Duncan J.M., Livesay J.J., Ott D.A., Gregoric I.D. (2006). Myocardial thievery: The coronary-subclavian steal syndrome. Ann. Thorac. Surg..

[38] Cua B., Mamdani N., Halpin D., Jhamnani S., Jayasuriya S., Mena-Hurtado C. (2017). Review of coronary subclavian steal syndrome. J. Cardiol..

[39] Uchida K., Karube N., Minami T., Cho T., Matsuki Y., Nemoto H., Yabu N., Yasuda S., Suzuki S., Masuda M. (2018). Treatment of coronary malperfusion in type A acute aortic dissection. Gen. Thorac. Cardiovasc. Surg..

[40] Kolozsvari R., Galajda Z., Ungvari T., Szabo G., Racz I., Szerafin T., Herzfeld I., Edes I., Peterffy A., Koszegi Z. (2012). Various clinical scenarios leading to development of the string sign of the internal thoracic artery after coronary bypass surgery: The role of competitive flow, a case series. J. Cardiothorac. Surg..

[41] Glineur D., Etienne P.-Y., Kuschner C.E., Shaw R.E., Ferrari G., Rioux N., Papadatos S., Brizzio M., Mindich B., Zapolanski A. (2017). Bilateral internal mammary artery Y construct with multiple sequential grafting improves survival compared to bilateral internal mammary artery with additional vein grafts: 10-year experience at 2 different institutions. Eur. J. Cardiothorac. Surg..

[42] Sabik J.F. (2011). Understanding saphenous vein graft patency. Circulation.

[43] Navia D.O., Vrancic M., Piccinini F., Camporrotondo M., Dorsa A., Espinoza J., Benzadon M., Camou J. (2016). Myocardial revascularization exclusively with bilateral internal thoracic arteries in T-graft configuration: Effects on late survival. Ann. Thorac. Surg..

[44] Borger M.A., Rao V., Weisel R.D., Ivanov J., Cohen G., Scully H.E., David T.E. (1998). Deep sternal wound infection: Risk factors and outcomes. Ann. Thorac. Surg..

[45] Peterson M.D., Borger M.A., Rao V., Peniston C.M., Feindel C.M. (2003). Skeletonization of bilateral internal thoracic artery grafts lowers the risk of sternal infection in patients with diabetes. J. Thorac. Cardiovasc. Surg..

[46] Puskas J.D., Sadiq A., Vassiliades T.A., Kilgo P.D., Lattouf O.M. (2012). Bilateral internal thoracic artery grafting is associated with significantly improved long-term survival, even among diabetic patients. Ann. Thorac. Surg..

[47] Deo S.V., Shah I.K., Dunlay S.M., Erwin P.J., Locker C., Altarabsheh S.E., Boilson B.A., Park S.J., Joyce L.D. (2013). Bilateral internal thoracic artery harvest and deep sternal wound infection in diabetic patients. Ann. Thorac. Surg..

[48] Lazar H.L. (2018). The risk of mediastinitis and deep sternal wound infections with single and bilateral, pedicled and skeletonized internal thoracic arteries. Ann. Cardiothorac. Surg..

[49] Galbut D.L., Kurlansky P.A., Traad E.A., Dorman M.J., Zucker M., Ebra G. (2012). Bilateral internal thoracic artery grafting improves long-term survival in patients with reduced ejection fraction: A propensity-matched study with 30-year follow-up. J. Thorac. Cardiovasc. Surg..

[50] Mohammadi S., Kalavrouziotis D., Cresce G., Dagenais F., Dumont E., Charbonneau E., Voisine P. (2014). Bilateral internal thoracic artery use in patients with low ejection fraction: Is there any additional long-term benefit?. Eur. J. Cardiothorac. Surg..

[51] Kainuma S., Toda K., Daimon T., Miyagawa S., Yoshikawa Y., Hata H., Yoshioka D., Kawamura T., Kawamura A., Kashiyama N. (2021). Bilateral internal thoracic artery grafting improves survival for severe left ventricular dysfunction and diabetes. Circ. J..

[52] Farkash A., Gordon A., Mohr R., Sela O., Pevni D., Ziv-Baran T., Grupper A., Kfir J.E., Ben-Gal Y. (2024). Single versus bilateral internal thoracic artery grafting in patients with impaired renal function. PLoS ONE.

[53] Navia D., Espinoza J., Vrancic M., Piccinini F., Camporrotondo M., Dorsa A., Seoane L. (2022). Bilateral internal thoracic artery grafting in elderly patients: Any benefit in survival?. J. Thorac. Cardiovasc. Surg..

[54] Kusu-Orkar T.-E., Kermali M., Masharani K., Noshirwani A., MacCarthy-Ofosu B., Oguamanam N., Bin Saeid J., Muir A.D., Harky A. (2021). Skeletonized or pedicled harvesting of left internal mammary artery: A systematic review and meta-analysis. Semin. Thorac. Cardiovasc. Surg..

[55] Kamiya H., Akhyari P., Martens A., Karck M., Haverich A., Lichtenberg A. (2008). Sternal microcirculation after skeletonized versus pedicled harvesting of the internal thoracic artery: A randomized study. J. Thorac. Cardiovasc. Surg..

[56] Benedetto U., Altman D.G., Gerry S., Gray A., Lees B., Pawlaczyk R., Flather M., Taggart D.P. (2016). ART Investigators. Pedicled and skeletonized single and bilateral internal thoracic artery grafts and the incidence of sternal wound complications: Insights from the Arterial Revascularization Trial. J. Thorac. Cardiovasc. Surg..

[57] Sá M.P., Ferraz P.E., Escobar R.R., Vasconcelos F.P., Ferraz A.A., Braile D.M., Lima R.C. (2013). Skeletonized versus pedicled internal thoracic artery and risk of sternal wound infection after coronary bypass surgery: Meta-analysis and meta-regression of 4817 patients. Interact. Cardiovasc. Thorac. Surg..

[58] Saha K.K. (2017). Graft spasm—The Achilles’ heel of arterial grafts. Indian Heart J..

[59] Saxena P., Mejia R., Tam R. (2005). Hydrodissection technique of harvesting left internal thoracic artery. Ann. Thorac. Surg..

[60] Chang S., Smith I., Cole C. (2025). Defining the cardiac surgical learning curve: A longitudinal cumulative analysis of a surgeon’s experience and performance monitoring in the first decade of practice. J. Cardiothorac. Surg..

[61] Gaudino M., Audisio K., Rahouma M., Chadow D., Cancelli G., Soletti G.J., Gray A., Lees B., Gerry S., Benedetto U. (2021). ART Investigators. Comparison of long-term clinical outcomes of skeletonized vs pedicled internal thoracic artery harvesting techniques in the Arterial Revascularization Trial. JAMA Cardiol..

[62] Jonsson A., Binongo J., Patel P., Wang Y., Garner V., Mitchell-Cooks D., Halkos M.E. (2023). Mastering the learning curve for robotic-assisted coronary artery bypass surgery. Ann. Thorac. Surg..

[63] Lamy A., Browne A., Sheth T., Zheng Z., Dagenais F., Noiseux N., Chen X., Bakaeen F.G., Brtko M., Stevens L.-M. (2021). Skeletonized vs pedicled internal mammary artery graft harvesting in coronary artery bypass surgery: A post hoc analysis from the COMPASS trial. JAMA Cardiol..

[64] Dalén M., Dismorr M., Glaser N., Sartipy U. (2024). Skeletonized versus pedicled harvesting of the internal thoracic artery and long-term clinical outcomes in coronary artery bypass surgery. J. Am. Heart Assoc..

[65] Sabik J.F., Lytle B.W., Blackstone E.H., Houghtaling P.L., Cosgrove D.M. (1999). Injury to a patent internal thoracic artery graft at coronary reoperation. Ann. Thorac. Surg..

[66] Beyls C., Huette P., Luang P., Kowalik H., Andriamifidy-Berti C., Guilbart M., Bernasinski M., Besserve P., Touati G., Caus T. (2025). Five-year mortality of patients with perioperative myocardial infarction after on-pump isolated or combined coronary artery bypass graft surgery: A retrospective propensity score-weighted analysis. J. Clin. Med..

[67] Balacumaraswami L., Taggart D.P. (2007). Intraoperative imaging techniques to assess coronary artery bypass graft patency. Ann. Thorac. Surg..

[68] Tokuda Y., Song M.H., Ueda Y., Usui A., Akita T. (2007). Predicting early coronary artery bypass graft failure by intraoperative transit time flow measurement. Ann. Thorac. Surg..

[69] ElBardissi A.W., Aranki S.F., Sheng S., O’Brien S.M., Greenberg C.C., Gammie J.S. (2012). Trends in isolated coronary artery bypass grafting: An analysis of the Society of Thoracic Surgeons adult cardiac surgery database. J. Thorac. Cardiovasc. Surg..

[70] Waterford S.D., Fischer C.P. (2024). Barriers to multiarterial coronary artery bypass grafting. Innovations.

[71] Little M., Gray A.M., Altman D.G., Benedetto U., Flather M., Gerry S., Lees B., Murphy J., Gaudino M., Taggart D.P. (2022). ART Investigators. Cost-effectiveness of bilateral vs. single internal thoracic artery grafts at 10 years. Eur. Heart J. Qual. Care Clin. Outcomes.

[72] Gaudino M., Rahouma M., Hameed I., Khan F.M., Taggart D.P., Flather M., Biondi-Zoccai G., Fremes S.E. (2019). Disagreement between randomized and observational evidence on the use of bilateral internal thoracic artery grafting: A meta-analytic approach. J. Am. Heart Assoc..

[73] Vrints C., Andreotti F., Koskinas K.C., Rossello X., Adamo M., Ainslie J., Banning A.P., Budaj A., Buechel R.R., Chiariello G.A. (2024). 2024 ESC Guidelines for the management of chronic coronary syndromes. Eur. Heart J..

